# The Sphygmometer, &c.

**Published:** 1835-07-01

**Authors:** 


*The Sphygmometer, &c. Being a Memoir presented to the Institute of France, by* Dr. Julius Hérisson; *with an Improvement of the Instrument, and Prefatory Remarks, by the Translator,* Dr. E. S. Blundell.—London, 1835. 8vo. pp. xvi. and 46.

The Sphygmometer, or pulse-measurer, is an instrument intended to measure the force of the pulse: its construction and use are described by Dr. Hérisson in the following passage.

"The instrument to which I have given the name of Sphygmometer, consists of a glass tube, three inches and a quarter in length, and two twelfths of an inch in diameter, graduated in front, and having a slip of coloured paper placed at the back with the graduated figures marked on it; the end of this glass tube is inserted into a steel stem, one inch and a quarter long, which is terminated at its base, by a hemi-spheroidal steel cup, three quarters of an inch in diameter, and two inches and one eighth in circumference, similar in form to an inverted tea cup, which is closed with a piece of gold-beater's skin stretched over its mouth, and confined by a rim in the same manner as parchment is secured on the head of a drum. All communication between the hemi-spheroidal cup, and the capillary duct of the stem and glass tube, is intercepted at will by a small stop-cock.

"There is in this hemi-spheroidal reservoir a certain quantity of mercury, which, whenever the instrument is properly applied in the course of an artery, receives and represents its action in the transparent tube. The same instrument on a larger scale serves to explore the heart. You will observe that the capillary duct, the hemi-spheroidal cup, and the quantity of mercury being the same in all Sphygmometers, they all possess the same power, and afford an identical measure of the pulse.

"When the pulse of an individual is to be examined, he may be seated or reclining on a couch; if he be seated, the physician places himself before the arm, the artery of which he is going to examine, and steadies this arm upon his left hand, or on his thigh, or on the arm of a chair; he holds the instrument by its base between the thumb and the index (fore) finger of his right hand, and applies it over the course of the radial artery in such a manner, that the artery shall correspond, as exactly as possible, to the centre of the reservoir. By pressing the artery (in the way above alluded to) with the right hand, the physician endeavours to discover the maximum of its impulse; and having ascertained this, he presses with the end of his thumb and index finger, upon the lateral parts of the artery, whose action is then transmitted to the column of mercury, which thus appears a mere continuation of the artery." (P. 3.)

The following table will show the indications afforded by the sphygmometer, in a number of cases of diseased heart.

| *Organic Lesions.* | *Character of the Pulse, and Sphygmometric Signs.* | *Necroscopic Examination.* | *Observations.* |
|---|---|---|---|
| "Auriculo-ventricular contractions at the right side of the heart, and auriculo-pulmonary contractions. — 22 cases. | Small, irregular, unequal, intermittent, and at times imperceptible. The column of mercury does not descend to the point from whence it started, or descends to it only in two periods. Near the middle it is surprised by an incidental impulsion. | Contractions of various kinds, and dilatations of the auricle and ventricle in a more or less forward state. A little hypertrophy was observed in the right ventricle of four individuals. | In eight of these patients, auscultation furnished only a slight *bruisement*. In six, the *bruit cataire* was distinctly marked; in the other eight, there was no abnormal sound. Oppression, and a more or less decided alteration of the features and colour of the face, were the only symptoms which might have raised any suspicions of the disease. Four patients died of pulmonary apoplexy; the rest in a state of general infiltration. |
| Auriculo-ventricular contractions on the left side of the heart, and ventriculo-aortic contractions.—27cases. | The pulse is feeble, irregular, intermittent, unequal, but much more so than in the contractions of the orifices on the right side. The column of mercury in the Shygmometer descends below its level, one, two, and even three degrees, according to the importance of the obstacle. | In twelve cases the heart was not affected with hypertrophy, but merely dilated. In the other fifteen there was a beginning of hypertrophy of the left auricle and ventricle. | In the first twelve, the pulse was extremely feeble: the patients died of hydrothorax, in a state of general infiltration. Of the remaining fifteen, eight sunk under hæmoptysis, five died of various affections of the lungs, and two of cerebral hæmorrhage. The pulse of these fifteen patients was hard, frequent, and brusque, but offered only a very inconsiderable development. |
| Hypertrophy of the heart, without any contraction of the orifices.—18 cases. | Pulse regular, but unequal in its contractions. It presents this anomaly, that the column of mercury, after having ascended a certain number of degrees, (we will say three or four,) rises suddenly by intervals up to eight, ten, and even fifteen degrees. | The autopsy of eighteen individuals, in whom I had observed the stated Sphygmometric sign, showed a concentric or excentric hypertrophy of the left ventricle, without any contraction of the orifices. | In those patients who laboured under a concentric hypertrophy, the pulse had not the same development as in excentric hypertrophy, but it presented the same character of inequality in its contractions. The signs derived from auscultation occurred in eight cases; in all the others they were so feebly marked, that it would have been impossible to recognize an advanced lesion of the heart by those signs." |

At page 37 we arrive at the extract of a report made to the Academy of Sciences, relative to the Sphygmometer of Dr.

Hérisson, by Messrs. Magendie and Serres. The report was unfavourable, and Dr. Hérisson has consequently thought fit to omit all the marrow, and give only a few skinny sentences. On this Dr. E. S. Blundell observes:

"Since translating the above, I have observed in the Athenæum of December 20th, 1834, p. 922, a notice of the report of Messrs. Majendie and Serres on the Sphygmometer, in which it is stated that these gentlemen gave it as their opinion, that its results are not more precise than those of the ordinary mode of feeling the pulse, since, 'having caused two persons, both equally skilled in the use of the Sphygmometer, to apply it successively to the radial artery of the same individual, and to write down separately the indications given by the instrument, the results obtained differed materially. I am entirely ignorant why Dr. Herisson has not given this objection in his abstract of the report, but, however this may be, it will be seen from the introductory page (xiii.) that a similar objection has already occurred to myself, and that I have, as I conceive, effectually obviated it by the improvement which I have made in Dr. Hérisson's instrument." (P. 40.)

Now, though we have not been so fortunate as to fall in with the report itself, yet we have been more lucky than our translator, as we have found a very long quotation from it in the American Journal of the Medical Sciences for February, 1835; and we shall transcribe the whole, as affording our readers a better means of arriving at Magendie's opinion than the garbled extracts of Dr. Hérisson; extracts which remind one rather of the disingenuous arts of a puffing advertiser, than the candour of a scientific physician.

"MM. Magendie and Serres, in their report to the Academy of Sciences, observe, 'If this contrivance were really competent to set before the eye the principal phenomena of the arterial circulation; if it could supply the means of measuring, and of course of expressing precisely, by signs similar to those by which variations of temperature, for example, are denoted; then would the sphygmometer, as it is called, prove an important acquisition in medicine; for even the most practised physician, no matter how delicate his sense of touch may be, is far from possessing, in his investigations by the pulse, a degree of certainty like that which results from the use of the thermometer. But it requires at least as much practice to learn the proper use of this instrument as it does to become acquainted with the ordinary indications of the pulse: nor are the results in any degree more exact. We have taken two persons, both familiar with the sphygmometer, and causing each successively to apply it to the radial artery of one and the same individual; we have requested them to write down separately what they ascertained by the instrument: the results, as stated by the parties, have been materially different, *(sensiblement divergents.)*

"The inventors of the sphygmometer, like most other inventors, promise and predict great things, as about to spring from their contrivance: they state, on the faith of facts which your reporters have not been able to verify, that the indications afforded by this instrument will furnish certain signs by which medical men may detect the presence of several maladies, whose diagnosis is at present obscure. While we are ready to admit that the sphygmometer is an ingenious thing, and not unworthy of trial in the hands of physicians, we can by no means partake of the sanguine hope of the authors: we moreover think that, if MM. Hérisson and P. Garnier intend to reach the object at which they aim, they must, by some modification in the apparatus, render its use more simple, and free from the necessity of conjectural approximations.

"In conclusion, the reporters propose that the thanks of the Academy be given to MM. Hérisson and Garnier, for their communication; and that those gentlemen be requested to simplify those instruments, if possible, so that the fidelity of its indications may no longer depend, as at present it does, on the cleverness and nice precautions of the observer.' Adopted."

We think that Dr. E. S. Blundell has been rather too hasty in publishing this translation: it would have been better to wait a few months, until the instrument had been proved in the hands of British physicians. This delay would also have afforded him time to procure an engraving representing his new apparatus; which he has not done, because, as he tells us, "considerable delay and expense would necessarily be incurred, greatly beyond the limits of the work." (P. 44.)

